# Geriatric nutritional risk index and body composition dictate the prognosis of elderly patients with intrahepatic cholangiocarcinoma

**DOI:** 10.3389/fnut.2025.1565317

**Published:** 2025-03-07

**Authors:** Sheng Wang, Luo Cheng, Lei Dou, Yuanli Kuang, Yang Huang, Tao Wen, Lei Xiang, Wenyuan Xie, Cheng Zhang, Dewei Li, Hui Li

**Affiliations:** ^1^Department of Hepatobiliary Pancreatic Tumor Center, Chongqing University Cancer Hospital, Chongqing, China; ^2^Department of General Surgery, Chongqing Kaizhou District People’s Hospital, Chongqing, China; ^3^Department of Liver Surgery, West China Hospital, Sichuan University, Chengdu, China

**Keywords:** intrahepatic cholangiocarcinoma, elderly patient, geriatric nutritional risk index, body composition, malnutrition

## Abstract

**Background and aims:**

Malnutrition is a well-recognized predictor of poor prognosis in malignancies. Recent studies suggest that the geriatric nutritional risk index (GNRI) is a more accurate determinant of prognosis in elderly patients than conventional body mass index (BMI). This study aimed to evaluate the GNRI and body composition parameters in elderly patients with intrahepatic cholangiocarcinoma (ICC) and assess their prognostic impact on long-term outcomes.

**Methods:**

A total of 157 elderly ICC patients (aged ≥65 years) who underwent radical resection between 2009 and 2018 were retrospectively analyzed. Skeletal muscle index (SMI), muscle attenuation (MA), visceral adipose tissue index (VATI), subcutaneous adipose tissue index (SATI), and visceral-to-subcutaneous fat ratio (VSR) were quantified using computed tomography. Prognostic analyses were conducted using the Kaplan–Meier method, with adjustments using inverse probability weighting. A nomogram based on multivariate Cox regression was constructed and internally validated, comparing its prognostic accuracy with the TNM staging system.

**Results:**

Among the body composition parameters, low SMI (sarcopenia, 56.1%), high VSR (visceral adiposity, 54.8%), and low MA (intramuscular fat deposition, 50.3%) were significantly associated with overall survival (OS) and recurrence-free survival (RFS) (all *p* < 0.05). Low GNRI was also a strong predictor of poor prognosis (*p* < 0.001). Multivariate analysis identified low GNRI (*p* = 0.009), sarcopenia (*p* = 0.020), visceral adiposity (*p* = 0.033), and intramuscular fat deposition (*p* = 0.036) as independent prognostic factors for OS and RFS. The nomogram, incorporating GNRI, SMI, VSR, MA, microvascular invasion (MVI), CA19-9 levels, and lymph node invasion, demonstrated superior prognostic performance compared to the TNM stage, with a C-index of 0.734 (OS) and 0.704 (RFS) and an AUC of 0.809 (OS) and 0.815 (RFS).

**Conclusion:**

GNRI, sarcopenia, IMF deposition, and visceral adiposity independently predict mortality and tumor recurrence in elderly ICC patients. Body composition is a major determinant of prognosis in patients with ICC. Our nomogram based on body composition reveals superior prognostic efficacy over TNM stages.

## Introduction

Liver cancer remains a major global health challenge, ranking as the third leading cause of cancer-related mortality worldwide (1). Intrahepatic cholangiocarcinoma (ICC) is a subset of liver cancer, which accounts for 10–15% of the cases (1). The incidence of ICC has increased over the past decades (2), especially among adults aged more than 60 years (3). Elderly cancer patients often experience higher surgical risks, impaired recovery, and increased mortality rates, largely due to malnutrition and age-related functional decline (4). They are not only devastated by disease but also persecuted by physical function impairment called geriatric syndrome. Such a proven relationship of malnutrition has been found in patients with hepatocellular carcinoma (5) and renal cell carcinoma (6).

Body mass index (BMI), a simple anthropometric index based on height and weight, is widely used for the assessment of nutrition status. However, it is limited due to insufficient in assessing individual components of body weight such as regional fat distribution or muscle volume (5). In addition, previous studies have shown that malnourished patients may not always have a low BMI, highlighting the need for more precise nutritional markers (7). Compared to BMI, the geriatric nutritional risk index (GNRI) is a more objective index for evaluating the nutritional status of patients aged ≥65 years. Previous studies have demonstrated its prognostic value in elderly patients with prostate cancer and heart failure (8). However, its significance in elderly ICC patients remains unclear.

Beyond nutrition, body composition plays a crucial role in cancer prognosis. Sarcopenia was first defined as a characterization of the age-related loss of skeletal muscle mass in 1989 (9). In 2010, the European Working Group on Sarcopenia in Older People (EWGSOP) clinically defined sarcopenia as a disease of progressive or systemic skeletal muscle, with a prevalence of approximately 10% in men and women aged more than 60 years (10). Emerging evidence has demonstrated that sarcopenia was linked to many negative consequences, such as falls, fractures, and even poor outcomes in many pathological conditions, which significantly strain the healthcare system (11). It is characterized by progressive and generalized loss of skeletal muscle mass and function with increasing age. It is a common but underappreciated complication of various chronic diseases and malignant tumors. Beyond skeletal muscle mass and quality, body composition, particularly the amount and distribution of adipose tissue, also plays a crucial role in sarcopenia. With aging, chronic adipose tissue inflammation contributes to the redistribution of subcutaneous fat to the intra-abdominal region (visceral adipose tissue), along with the infiltration of fat into skeletal muscles (12).

Therefore, this study aimed to investigate the prognostic value of the GNRI in combination with key body composition parameters (skeletal muscle and adipose tissue) in a retrospective cohort of 157 elderly patients with ICC. In addition, we sought to develop a comprehensive prognostic model to enhance risk stratification and improve clinical decision-making for this patient population.

## Methods

### Study population

A total of 528 ICC patients who underwent radical resection at Chongqing University Cancer Hospital (Chongqing, China) and West China Hospital (Chengdu, China) from 2009 to 2018 were retrospectively reviewed. Elderly patients were defined as those aged over 65 years. The exclusion criteria were as follows: preoperative radiofrequency ablation, transarterial chemoembolization, or other anti-cancer therapies; extrahepatic metastasis; patients underwent liver transplantation or surgical resection for tumor rupture; patients without imaging data. Patients who met the exclusion criteria were included in the analysis. The collected data included patient demographics, clinicopathological characteristics, preoperative computed tomography (CT) images, and disease status at the end of follow-up. This study was performed in accordance with the guidelines of the 1975 Declaration of Helsinki.

### Follow-up

Surgical procedures were determined based on preoperative multidisciplinary discussions and intraoperative evaluations. Lymphadenectomy for ICC was performed according to the recommendations by Shimada et al. (13). The follow-up strategy was conducted in accordance with the National Comprehensive Cancer Network (NCCN) guidelines. In brief, the tumor marker carbohydrate antigen 19-9 (CA19-9) and imaging examinations (ultrasonography or CT) were performed monthly during the first 3 months, then every 3 months for 1 year, and subsequently every 6 months thereafter. For patients who were unable to return to the hospital for reexamination, telephone follow-up surveys were conducted to collect relevant clinical data. Overall survival (OS) was defined as the duration from the date of hepatic resection to death or the last follow-up. Recurrence-free survival (RFS) was defined as the period from the date of surgery to the first documented recurrence or the last follow-up.

### Body composition

As previously described (4), GNRI was calculated using the formula: GNRI = 14.89 × serum albumin (g/dL) + 41.7 × [present body weight (kg)/ideal body weight (kg)]. The ideal weight was calculated by 22 × [height (m)]^2^. Cross-sectional non-contrast plain CT images were applied for the assessment of body composition using Mimics software (version 21.0, Materialise NV, Leuven, Belgium). The measurements were validated by experienced radiologists. The areas of skeletal muscle and abdominal adipose tissue were determined at the level of the third lumbar vertebra. Skeletal muscle areas included the psoas, quadratus lumborum, erector spinae, transversus abdominis, and rectus abdominis. Tissue Hounsfield unit (HU) thresholds were used as previously reported (5): −29 to 150 HU for skeletal muscle, −150 to −50 for visceral adipose tissue, and −190 to −30 for subcutaneous adipose tissue. We termed the parameters for skeletal muscle as skeletal muscle index (SMI), visceral adipose tissue as visceral adipose tissue index (VATI), and subcutaneous adipose tissue as subcutaneous adipose tissue index (SATI), respectively. SMI was calculated as cross-sectional area of skeletal muscle (cm^2^)/height^2^ (m^2^). Visceral-to-subcutaneous adipose tissue area ratio (VSR) was calculated to determine abdominal adipose tissue distributions. In addition, muscle attenuation (MA) was applied for the assessment of skeletal muscle quality (14).

### Statistical analysis

The continuous variable was expressed as mean ± standard deviation (SD) and analyzed using Student’s *t*-test or Wilcoxon rank sum test, depending on data distribution. Categorical variables were analyzed using the chi-square test or Fisher’s exact test, as appropriate. The optimal cutoff values for sex-specific GNRI and body composition variables were determined using receiver operating characteristic (ROC) curves, selecting the threshold at which the Youden index reached its maximum value. Kaplan–Meier (K–M) curves were plotted to illustrate cumulative mortality and recurrence, with differences tested using the log-rank test. Univariate and multivariate Cox proportional hazard models were used to identify independent prognostic indicators. Variables with a *p*-value of <0.05 in univariate analysis were included in the multivariate analysis. A prognostic nomogram was developed using independent prognostic factors identified through multivariate analysis, utilizing a backward stepwise selection method. The concordance index (C-index) and area under the curve (AUC) of time-dependent receiver operating characteristic (td-ROC) curves were used to compare the predictive performance of the nomogram and the conventional tumor-node-metastasis (TNM) staging system. The integrated AUC was calculated as the average AUC across different time points. Statistical analyses were performed by SPSS (version 23.0, Chicago, IL, United States), MedCalc (version 20.0.3.0, Ostend, Belgium), and R software (version 4.1.0).^1^ A two-sided *p*-value of <0.05 was considered statistically significant.

## Results

### Patient characteristics

Among 528 ICC patients from a multi-institutional database, 157 met the inclusion criteria for this study (Supplementary Figure S1). The mean age was 70.1 years, and 74 patients (47.1%) were male. HBV infection was observed in 23 patients (14.6%), and 32 patients (20.4%) had hepatolithiasis. CA19-9 levels were within the normal range in 38 patients (24.2%). Regarding tumor characteristics, 122 patients (77.7%) presented with a solitary tumor, with a mean tumor size of 5.6 cm. The majority (116, 73.9%) were classified as TNM stage III.

The median BMI was 22 kg/m^2^ (IQR: 20.2–24.1). The median GNRI was 102.3 (IQR: 98.1–109.3) in males and 103.7 (IQR: 95.8–110.5) in females. The median SMI was 39.5 (IQR: 36.3–42.7) for males and 35.9 (IQR: 32.7–39.6) for females. Further baseline characteristics are provided in Table 1.

**Table 1:** Baseline characteristics of included ICC patients.

Variables	*N* = 157
Age, year	70.1 ± 4.2
Male gender, *n* (%)	74 (47.1)
Body mass index (kg/m^2^), median (IQR)	22.2 (20.2–24.1)
Smoking status, *n* (%)	
Never/former/current	64 (40.7)/51 (32.5)/42 (26.8)
HBsAg positive, *n* (%)	23 (14.6)
Hepatolithiasis, *n* (%)	32 (20.4)
CA19-9 < 37, *n* (%)	38 (24.2)
Tumor size, cm	5.6 (2.5)
Solitary tumor, *n* (%)	122 (77.7)
Cirrhosis, *n* (%)	27 (17.2)
Well tumor differentiation, *n* (%)	56 (35.7)
Capsule invasion, *n* (%)	103 (65.6)
Perineural invasion, *n* (%)	27 (17.2)
Number of LNs reviewed, mean (SD)	4.3 (2.6)
Lymph node invasion, *n* (%)	29 (18.5)
MVI, *n* (%)	15 (9.6)
TNM stage, *n* (%)	
I–II/III	41 (26.1)/116 (73.9)
GNRI, median (IQR)	102.7 (96.2–109.4)
Male/female	102.3 (98.1–109.3)/103.7 (95.8–110.5)
SMI (cm^2^/m^2^), median (IQR)	37.5 (33.9–41.1)
Male/female	39.5 (36.3–42.7)/35.9 (32.7–39.6)
VATI (cm^2^/m^2^), median (IQR)	34.6 (28.4–40.1)
Male/female	36.2 (30.1–41.2)/30.8 (24.6–38.7)
SATI (cm^2^/m^2^), median (IQR)	37.2 (29.4–47.3)
Male/female	33.8 (28.6–44.1)/43.4 (30.2–51.3)
VSR, median (IQR)	0.73 (0.59–0.97)
Male/female	0.92 (0.69–1.13)/0.63 (0.53–0.79)
MA (HU), median (IQR)	39.1 (34.3–45.5)
Male/female	43.3 (36.6–47.7)/37.1 (31.6–43.2)

ICC, intrahepatic cholangiocarcinoma; CA19-9, carbohydrate antigen 19-9; MVI, microvascular invasion; GNRI, geriatric nutritional risk index; SMI, skeletal muscle index; VATI, visceral adipose tissue index; SATI, subcutaneous adipose tissue index; VSR, visceral-to-subcutaneous fat area ratio; MA, muscle attenuation.

### Association between GNRI, body compositions, and prognosis of elderly ICC patients

**Figure 1:**
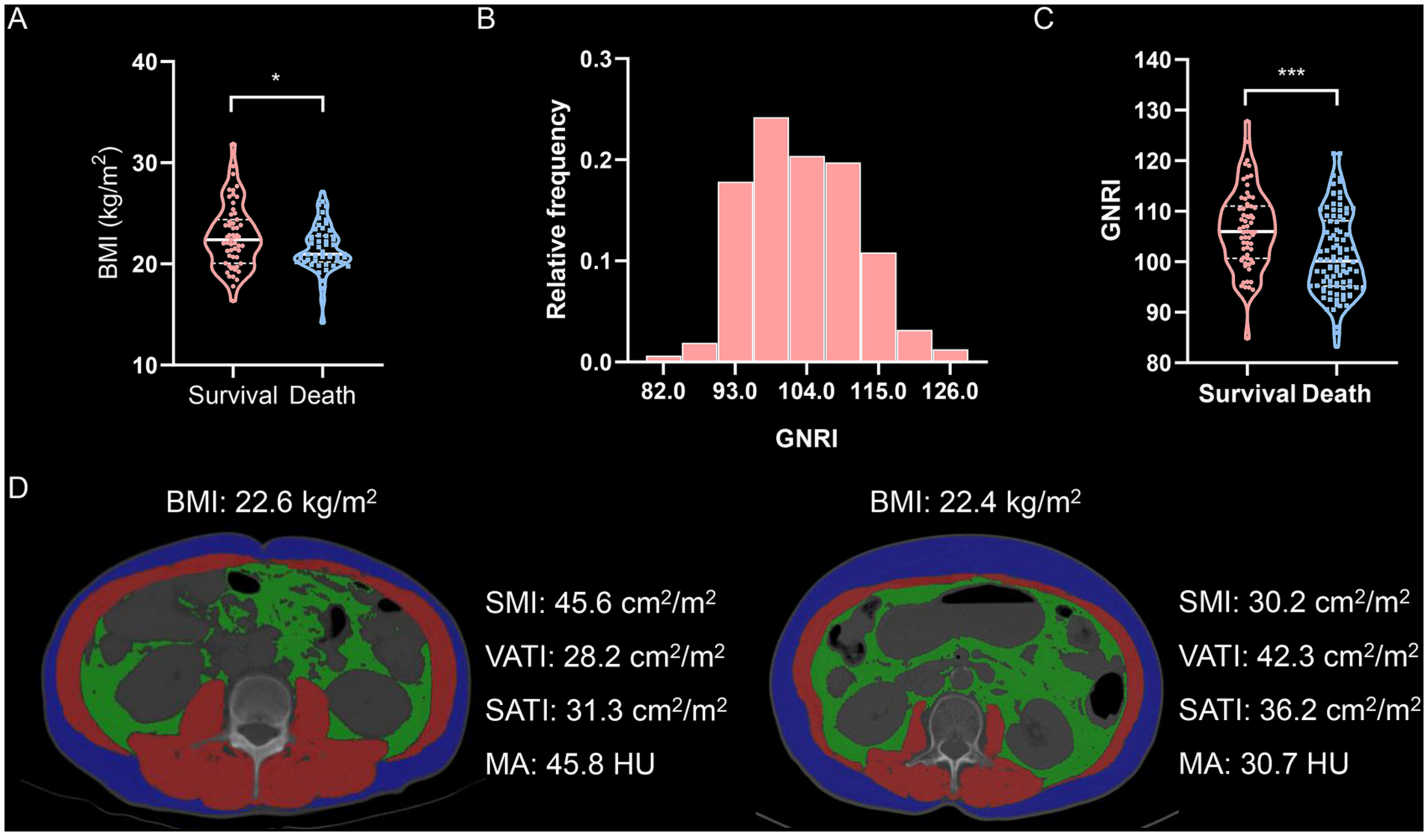
Body compositions quantified by cross-sectional computed tomography images of the third lumbar vertebra. **(A)** Violin plot shows BMI values in survival or death. **(B)** Distribution of GNRI values. **(C)** Violin plot shows GNRI values in survival or death. **(D)** Cross-sectional computed tomographic images at the level of the third lumbar vertebra among two patients with almost identical BMI (The red shadows represent the skeletal muscle areas, the green shadows represent the visceral adipose tissue areas, and the blue shadows represent the subcutaneous adipose tissue areas, respectively.). GNRI, geriatric nutritional risk index; SMI, skeletal muscle index; VATI, visceral adipose tissue index; SATI, subcutaneous adipose tissue index; MA, muscle attenuation.

First, we examined the relationship between body mass index (BMI) and mortality in elderly intrahepatic cholangiocarcinoma (ICC) patients. Patients who survived had a higher BMI compared to those who did not (Figure 1A). However, Kaplan–Meier survival analysis revealed that underweight (BMI < 20 kg/m^2^), normal weight, and overweight (BMI > 24 kg/m^2^) patients had comparable overall survival (OS) and tumor relapse rates (Supplementary Figure S2). These findings suggest that BMI alone is not significantly associated with mortality in elderly ICC patients.

Next, we analyzed the geriatric nutritional risk index (GNRI) and four indicators of body composition—skeletal muscle index (SMI), visceral adipose tissue index (VATI), subcutaneous adipose tissue index (SATI), and muscle attenuation (MA)—using cross-sectional non-contrast CT images. GNRI was significantly lower in deceased patients compared to survivors (Figures 1B,C). The optimal GNRI cutoff values were determined to be 107.3 for males and 108.2 for females. BMI showed only a weak correlation with GNRI (Supplementary Figure S3A). Moreover, SMI, visceral-to-subcutaneous fat ratio (VSR), and MA exhibited no clear correlation with BMI (Supplementary Figure S3B–D), indicating that BMI alone is not a reliable measure for assessing body composition. Notably, two patients with similar BMI values demonstrated substantial differences in body composition (Figure 1D). Further correlation analysis revealed that GNRI was moderately associated with SMI, VSR, and MA (Supplementary Figure S4).

**Figure 2:**
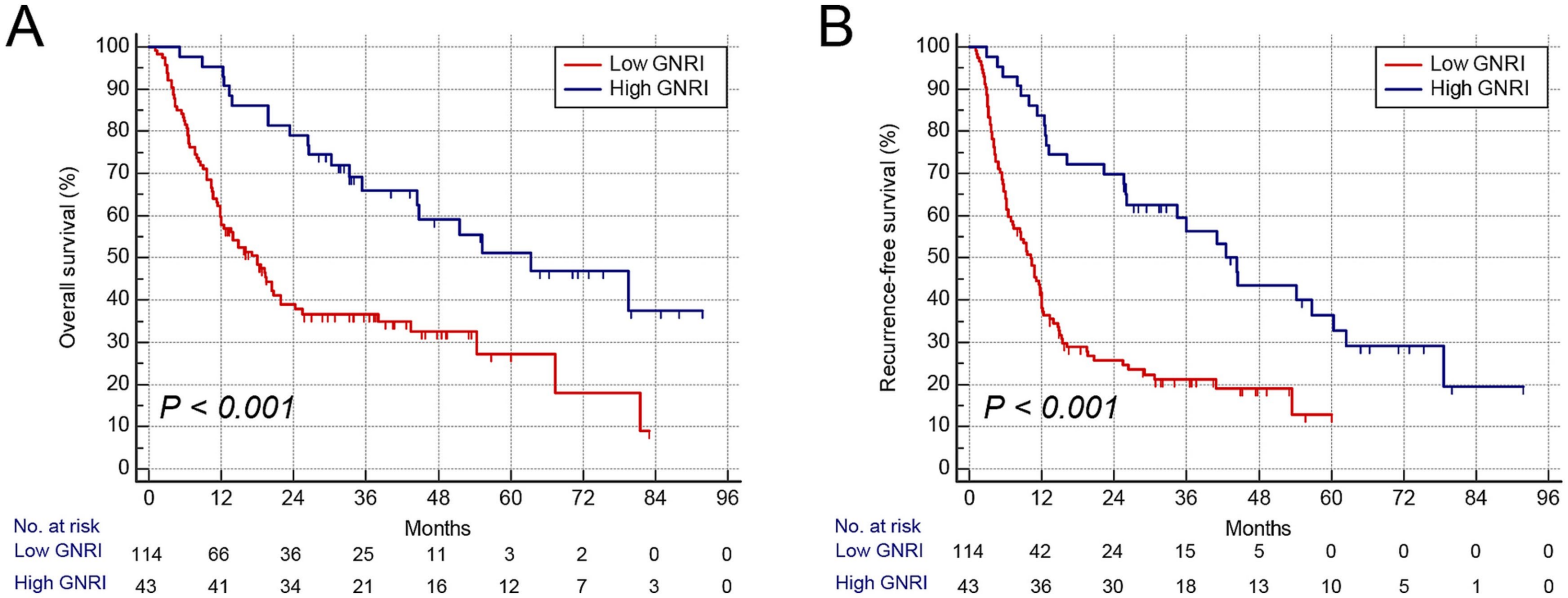
Kaplan–Meier curves showed overall and recurrence-free survival according to GNRI. **(A)** Overall survival rate after surgery classified by GNRI. **(B)** Recurrence-free survival rate after surgery classified by GNRI. GNRI, geriatric nutritional risk index.

**Figure 3:**
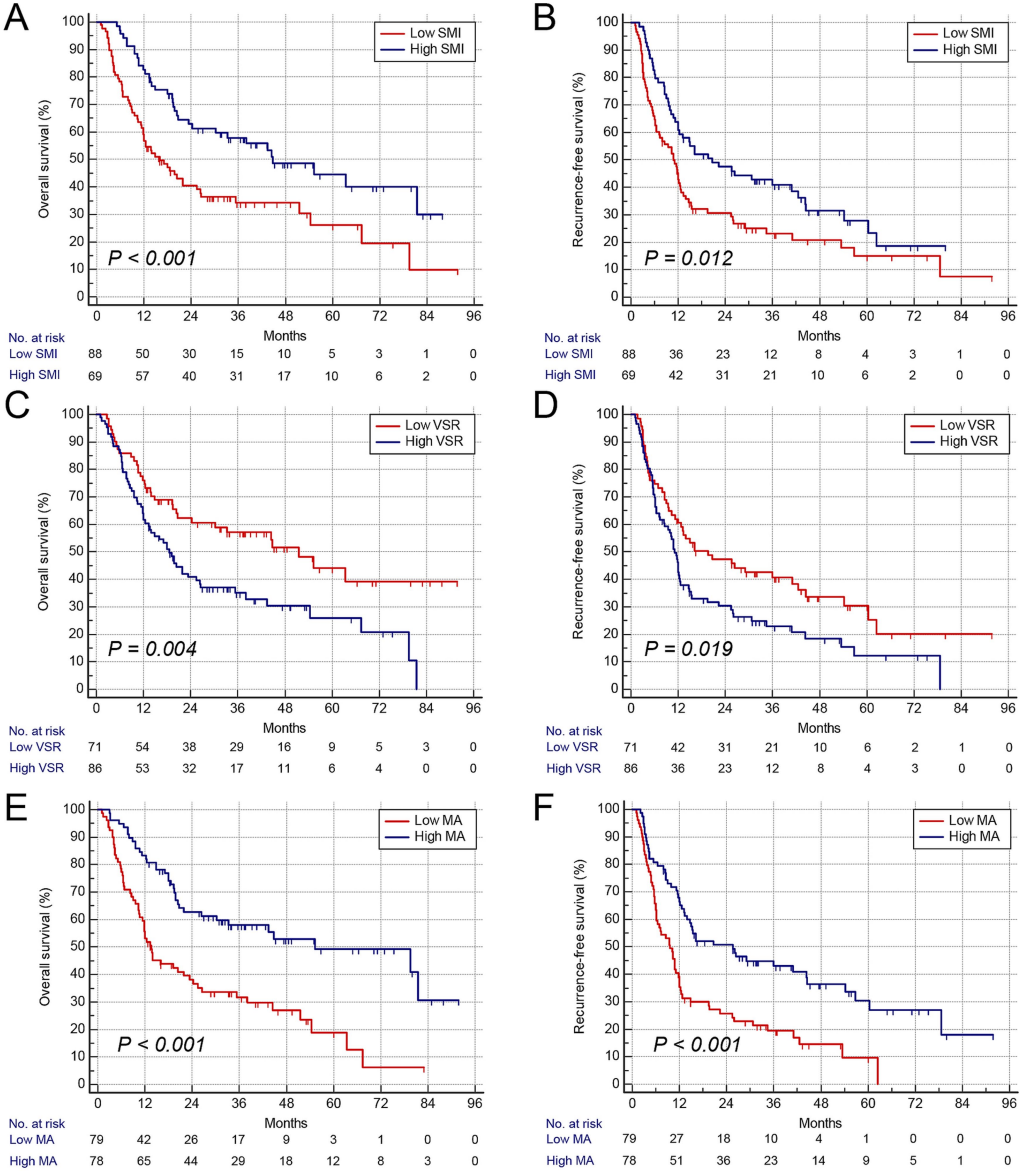
Kaplan–Meier curves show overall and recurrence-free survival according to body composition variables. **(A)** Overall survival rate after surgery classified by SMI. **(B)** Recurrence-free survival rate after surgery classified by VSR. **(C)** Overall survival rate after surgery classified by GNRI. **(D)** Recurrence-free survival rate after surgery classified by VSR. **(E)** Overall survival rate after surgery classified by MA. **(F)** Recurrence-free survival rate after surgery classified by MA. SMI, skeletal muscle index; VSR, visceral-to-subcutaneous fat area ratio; MA, muscle attenuation.

Consistent with GNRI, gender-specific cutoff values were determined for these body composition parameters (Supplementary Figure S5). The cutoff values for SMI were 41.3 cm^2^/m^2^ in males and 37.2 cm^2^/m^2^ in females, those for VSR were 1.04 in males and 0.87 in females, and those for MA were 42.3 HU in males and 38.1 HU in females, respectively. Patients with low SMI were classified as having sarcopenia, those with high VSR as having visceral adiposity, and those with low MA as having intramuscular fat (IMF) deposition. Furthermore, we analyzed the association between these parameters and prognosis using Kaplan–Meier survival curves. Patients with low GNRI (*n* = 114) exhibited significantly higher mortality and earlier postoperative tumor relapse (Figures 2A,B). Similarly, patients with sarcopenia (*n* = 88), visceral adiposity (*n* = 86), and IMF deposition (*n* = 79) demonstrated shorter OS and recurrence-free survival (RFS) (Figures 3A–F). Collectively, these results indicate that both GNRI and body composition factors are prognostic indicators in elderly ICC patients following curative resection.

**Figure 4:**
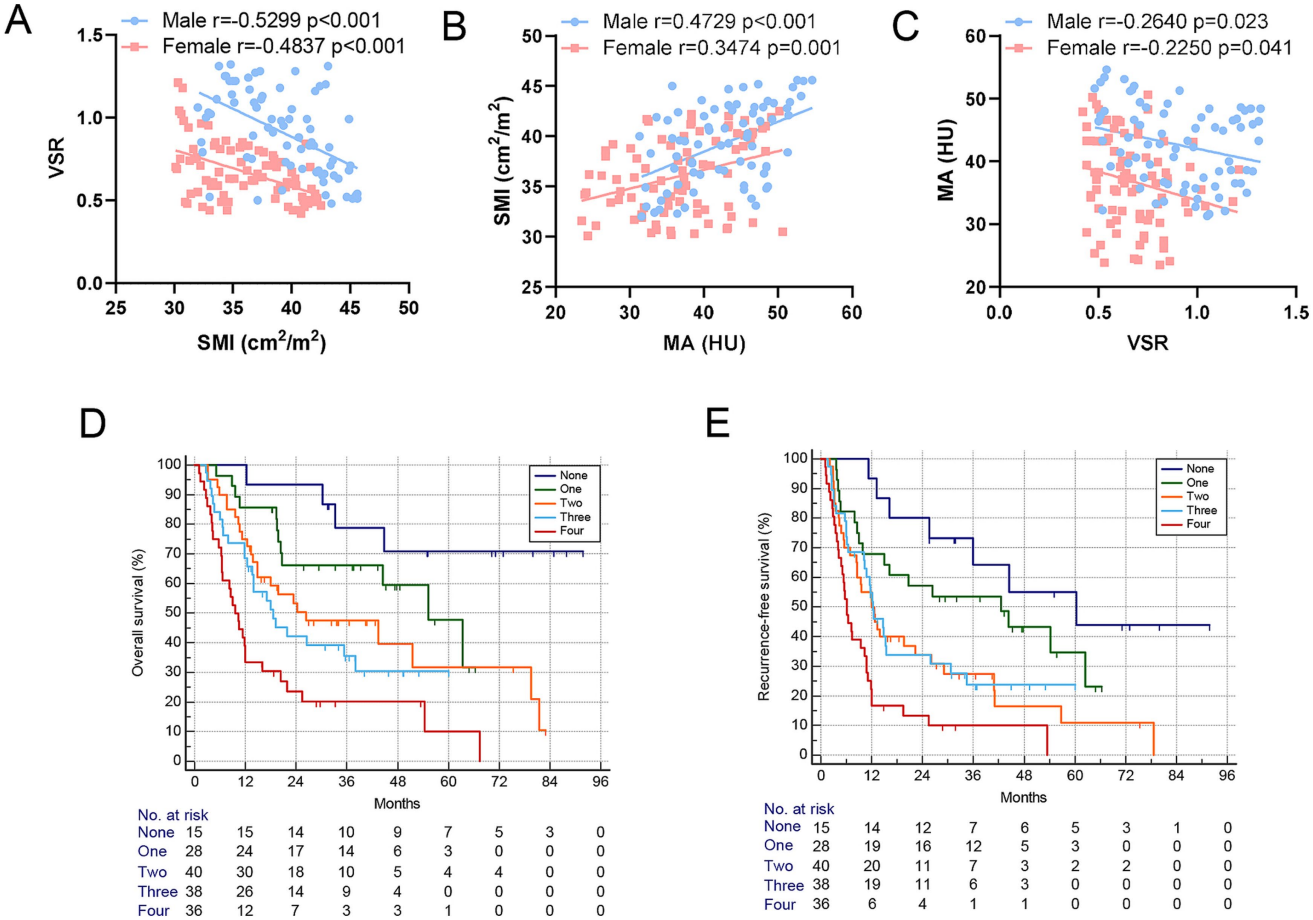
Body composition components served as complementary prognostic indicators for elderly ICC patients. **(A–C)** Correlation between SMI, VSR, and MA shows a significant correlation between them in both men and women. **(D,E)** Kaplan–Meier curves show a decreased overall survival rate and increased postoperative recurrence frequency based on the increase in the number of GNRI and body composition components. GNRI, geriatric nutritional risk index; SMI, skeletal muscle index; VSR, visceral-to-subcutaneous adipose tissue area ratio; MA, muscle attenuation.

Table 2 summarizes the clinicopathological characteristics of elderly ICC patients stratified by GNRI, sarcopenia, visceral adiposity, and IMF deposition. Notably, patients with low GNRI, sarcopenia, and IMF deposition had significantly larger tumor sizes. Moreover, perineural invasion and lymph node invasion were more frequently observed in patients with low GNRI and IMF deposition. Scatterplots demonstrated significant correlations among SMI, VSR, and MA (Figures 4A–C). Importantly, GNRI and these three body composition parameters collectively contributed to an increased risk of mortality and tumor recurrence in an additive manner (both *p* < 0.05, Figures 4D–E), suggesting that they serve as complementary prognostic markers for elderly ICC patients.

**Table 2:** Clinicopathological characteristics in 157 elderly ICC patients stratified by GNRI, SMI, VSR, and MA.

Variables	GNRI			SMI			VSR			MA		
	Low	High	*p*-value	Low	High	*p*-value	Low	High	*p*-value	Low	High	*p*-value
Case number	114	43		88	69		71	86		79	78	
Age, year	69.9 (4.5)	70.5 (3.4)	0.487	69.9 (4.5)	70.3 (3.9)	0.536	69.7 (4.0)	70.4 (4.4)	0.365	69.3 (3.9)	70.8 (4.4)	0.024
Male gender, *n* (%)	57 (50.0)	17 (39.5)	0.284	41 (46.6)	33 (47.8)	0.878	34 (47.9)	40 (46.5)	0.874	41 (51.9)	33 (42.3)	0.264
Body mass index (kg/m^2^), median (IQR)	21.9 (20.2–23.4)	23.4 (21.2–26.4)	0.031	22.1 (20.2–24.4)	22.5 (20.6–24.0)	0.718	22.4 (20.1–24.1)	22.3 (20.3–22.8)	0.817	22.8 (20.0–25.6)	22.7 (20.7–25.2)	0.932
Current smoking, *n* (%)	29 (25.9)	13 (31.0)	0.547	22 (25.6)	20 (29.4)	0.716	23 (33.3)	19 (22.4)	0.148	21 (26.9)	21 (27.6)	0.921
HBsAg positive, *n* (%)	17 (15.0)	6 (14.0)	0.864	12 (13.6)	11 (16.2)	0.820	11 (15.7)	12 (14.0)	0.822	10 (12.8)	13 (16.7)	0.652
Hepatolithiasis, *n* (%)	27 (23.7)	5 (11.6)	0.121	18 (20.5)	14 (20.3)	0.980	14 (19.7)	18 (20.9)	0.851	18 (22.8)	14 (17.9)	0.553
CA19-9 < 37, *n* (%)	23 (20.5)	15 (34.9)	0.088	19 (21.8)	19 (27.9)	0.129	24 (34.8)	14 (16.3)	0.015	18 (23.1)	20 (26.0)	0.552
Tumor size, cm	6.1 (2.7)	4.9 (2.8)	0.012	6.5 (2.9)	4.9 (2.4)	0.001	5.4 (2.7)	6.1 (2.8)	0.091	6.5 (2.9)	5.1 (2.5)	0.002
Solitary tumor, *n* (%)	89 (78.1)	33 (76.7)	0.859	66 (75.0)	56 (81.2)	0.441	51 (71.8)	71 (82.6)	0.125	59 (74.7)	63 (80.8)	0.444
Cirrhosis, *n* (%)	18 (15.8)	9 (20.9)	0.480	13 (14.8)	14 (20.3)	0.399	12 (16.9)	15 (17.4)	0.929	15 (19.0)	12 (15.4)	0.673
Well tumor differentiation, *n* (%)	39 (34.2)	17 (39.5)	0.578	27 (30.7)	29 (42.0)	0.179	31 (43.7)	25 (29.1)	0.067	27 (34.2)	29 (37.2)	0.741
Capsule invasion, *n* (%)	79 (69.3)	24 (55.8)	0.133	59 (67.0)	44 (63.8)	0.736	44 (62.0)	59 (68.6)	0.403	50 (63.3)	53 (67.9)	0.615
Perineural invasion, *n* (%)	25 (21.9)	2 (4.7)	0.016	15 (17.0)	12 (17.4)	0.955	8 (11.3)	19 (22.1)	0.090	20 (25.3)	7 (9.0)	0.010
Lymph node invasion, *n* (%)	26 (22.8)	3 (7.0)	0.036	21 (23.9)	8 (11.6)	0.062	15 (21.1)	14 (16.3)	0.536	20 (25.4)	9 (11.5)	0.039
MVI, *n* (%)	12 (10.5)	3 (7.0)	0.565	10 (11.4)	5 (7.2)	0.426	6 (8.5)	9 (10.5)	0.788	10 (12.7)	5 (6.4)	0.277
TNM stage III, *n* (%)	90 (78.9)	26 (60.5)	0.025	69 (78.4)	47 (68.1)	0.200	50 (70.4)	66 (76.7)	0.466	60 (75.9)	56 (71.8)	0.589

ICC, intrahepatic cholangiocarcinoma; CA19-9, carbohydrate antigen 19-9; MVI, microvascular invasion; GNRI, geriatric nutritional risk index; SMI, MI, skeletal muscle index; VSR, visceral-to-subcutaneous fat area ratio; MA, muscle attenuation.

### Establishment of a prognostic nomogram

Cox regression analyses were performed to identify independent risk factors associated with mortality and postoperative tumor recurrence (Table 3). Univariate analysis identified abnormal CA19-9 levels, poor tumor differentiation, perineural invasion, lymph node invasion, MVI, low GNRI, sarcopenia, visceral adiposity, and IMF deposition as potential predictors of poor OS and RFS. However, in multivariate analysis, tumor differentiation and perineural invasion were no longer significant prognostic factors. In contrast, abnormal CA19-9 levels, MVI, lymph node invasion, low GNRI, sarcopenia, visceral adiposity, and IMF deposition remained independent risk factors for poor prognosis in elderly ICC patients. In addition, multiple tumor number was identified as an independent risk factor for earlier tumor relapse.

**Table 3:** Prognostic factor analysis for overall survival and recurrence-free survival.

Variables	Overall survival				Recurrence-free survival			
	Univariate analysis		Multivariate analysis		Univariate analysis		Multivariate analysis	
	HR (95% CI)	*p*-value	HR (95% CI)	*p*-value	HR (95% CI)	*p*-value	HR (95% CI)	*p*-value
Age	0.984 (0.935–1.037)	0.550			0.957 (0.913–1.004)	0.073		
Gender	1,381 (0.920–2.073)	0.119			1.228 (0.853–1.768)	0.270		
Body mass index	0.965 (0.885–1.053)	0.424			0.998 (0.929–1.074)	0.967		
Smoking status	1.291 (0.830–2.007)	0.257			0.948 (0.628–1.430)	0.798		
HBsAg positive	1.323 (0.781–2.243)	0.298			1.298 (0.791–2.130)	0.302		
Hepatolithiasis	1.427 (0.883–2.304)	0.146			1.183 (0.753–1.861)	0.466		
CA19-9, ≥37/< 37	2,178 (1.552–3.057)	<0.001	1.815 (1.252–2.630)	0.002	1.556 (1.153–2.099)	0.004	1.915 (1.304–2.812)	0.001
Tumor size, ≥5/< 5	1.330 (0.884–2.001)	0.171			1.460 (1.007–2.116)	0.046	1.076 (0.682–1.698)	0.754
Multiple tumor	1.357 (0.852–2.159)	0.199			1.577 (1.043–2.385)	0.031	1.563 (1.016–2.404)	0.042
Cirrhosis, *n* (%)	1.495 (0.899–2.485)	0.121			1.262 (0.791–2.013)	0.329		
Tumor differentiation	1.668 (1.074–2.590)	0.023	1.409 (0.875–2.267)	0.158	1.579 (1.064–2.343)	0.023	1.381 (0.859–2.219)	0.182
Capsule invasion, *n* (%)	1.017 (0.664–1.555)	0.940			1.168 (0.792–1.723)	0.432		
Perineural invasion, *n* (%)	2.498 (1.521–4.102)	<0.001	1.338 (0.781–2.294)	0.289	1.719 (1.074–2.752)	0.024	1.565 (0.871–2.811)	0.134
Lymph node invasion, *n* (%)	2.999 (1.871–4.809)	<0.001	2.030 (1.183–3.484)	0.010	2.235 (1.440–3,470)	<0.001	1.840 (1.059–3.197)	0.030
MVI, *n* (%)	1.970 (1.042–3.724)	0.037	1.598 (1.011–2.527)	0.045	1.791 (1.001–3.207)	0.048	1.428 (0.723–2.818)	0.305
TNM stage, III/I-II	1.248 (0.935–1.666)	0.132			1.544 (0.997–2.390)	0.052		
GNRI, high/low	0.382 (0.230–0.635)	<0.001	0.497 (0.293–0.843)	0.009	0.355 (0.223–0.567)	<0.001	0.490 (0.286–0.840)	0.009
SMI, high/low	0.499 (0.327–0.761)	0.001	0.595 (0.384–0.923)	0.020	0.621 (0.427–0.901)	0.012	0.617 (0.395–0.963)	0.033
VSR, high/low	1.857 (1.215–2.838)	0.004	1.610 (1.040–2.493)	0.033	1.561 (1.074–2.268)	0.019	1.610 (1.040–2.493)	0.033
MA, high/low	0.407 (0.267–0.620)	<0.001	0.609 (0.383–0.968)	0.036	0.477 (0.327–0.696)	<0.001	0.544 (0.343–0.864)	0.010

ICC, intrahepatic cholangiocarcinoma; CA19-9, carbohydrate antigen 19-9; MVI, microvascular invasion; GNRI, geriatric nutritional risk index; SMI, MI, skeletal muscle index; VSR, visceral-to-subcutaneous fat area ratio; MA, muscle attenuation.

**Figure 5:**
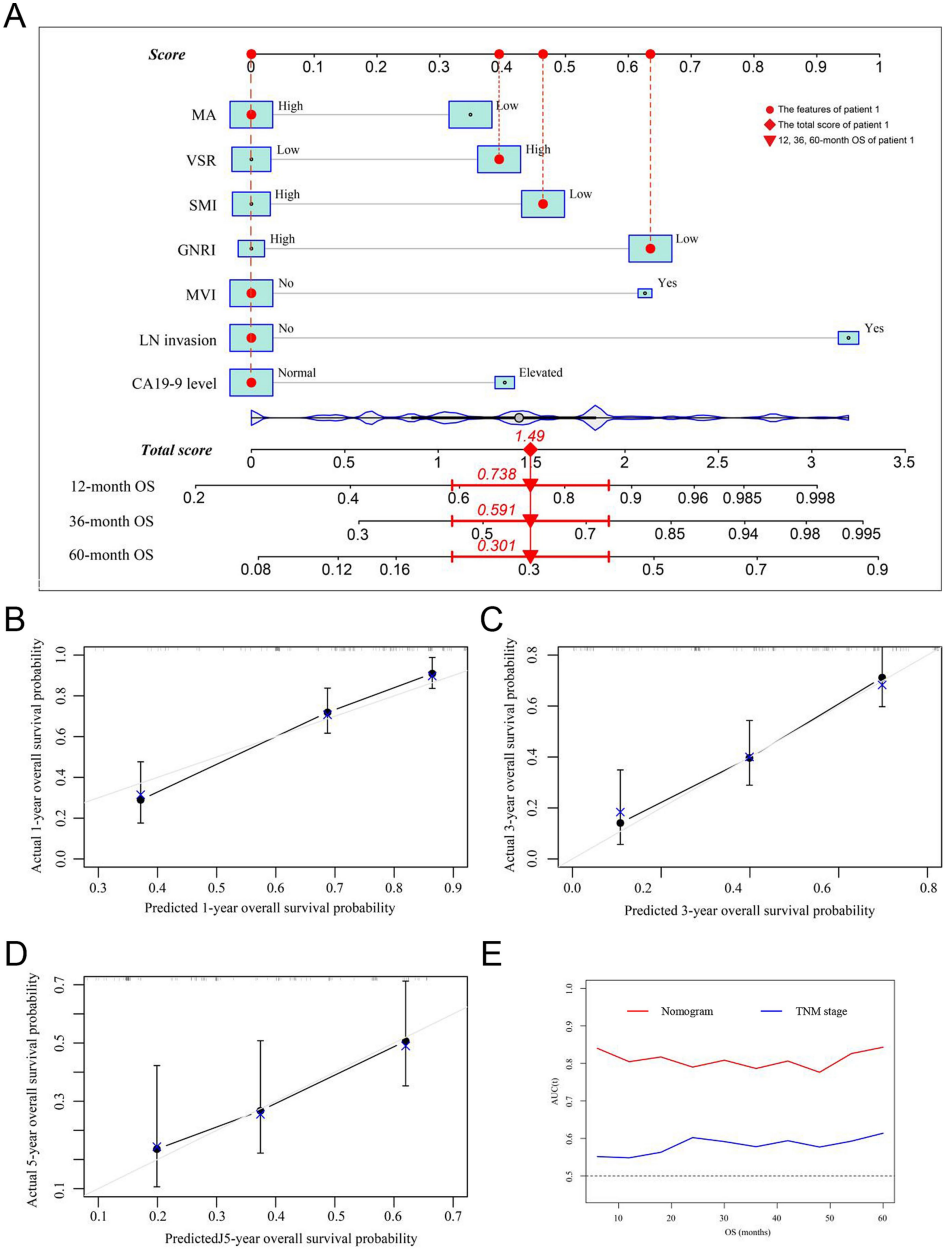
Establishment and validation of the prognostic nomogram for OS. **(A)** Nomogram was established using independent risk factors of OS. Each score was added up to obtain a total score. Patient 1 from this study was shown as an example (marked in red sign). **(B–D)** Calibration plots at 1-, 3-, and 5-year prediction showed the stabilization of the prognostic nomograms. **(E)** Time-dependent ROC compared the accuracy of the nomogram and AJCC-TNM stage in predicting OS showed better performance of the prognostic nomogram. OS, overall survival; AUC, area under the curve; ROC, receiver operating characteristic. GNRI, geriatric nutritional risk index; SMI, skeletal muscle index; VSR, visceral-to-subcutaneous adipose tissue area ratio; MA, muscle attenuation.

**Figure 6:**
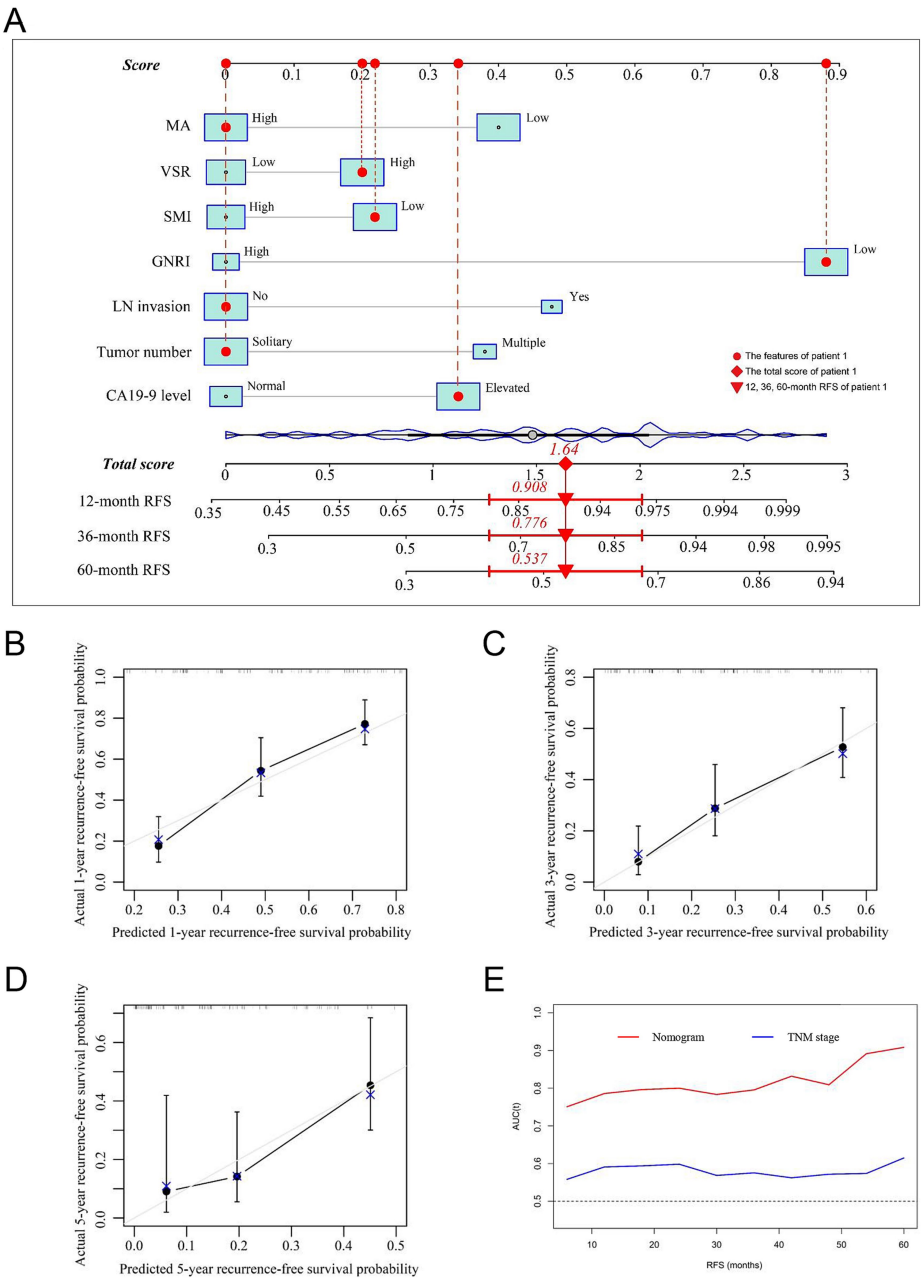
Establishment and validation of the prognostic nomogram for tumor relapse. **(A)** Nomogram was established using independent risk factors of RFS. Each score was added up to obtain a total score. Patient 1 from this study was shown as an example (marked in red sign). **(B–D)** Calibration plots at 1-, 3-, and 5-year prediction show the stabilization of the prognostic nomograms. **(E)** Time-dependent ROC compared the accuracy of nomogram and AJCC-TNM stage in predicting RFS showed better performance of the prognostic nomogram. RFS, recurrence-free survival; AUC, area under the curve; ROC, receiver operating characteristic. GNRI, geriatric nutritional risk index; SMI, skeletal muscle index; VSR, visceral-to-subcutaneous adipose tissue area ratio; MA, muscle attenuation.

Prognostic nomograms were developed based on the independent factors identified in the multivariate analysis. As shown in Figure 5A, the nomogram for OS achieved a C-index of 0.734 (95% CI: 0.697–0.773). The AUC values at 1, 3, and 5 years, along with the integrated AUC, were 0.805, 0.786, 0.843, and 0.809, respectively (Table 4). Calibration plots demonstrated strong concordance between the predicted and actual survival rates for OS (Figures 5B–D). Similarly, the nomogram for RFS showed a promising prognostic performance, with a C-index of 0.704 (95% CI: 0.669–0.739) (Figure 6A). The AUC values at 1, 3, and 5 years, along with the integrated AUC, were 0.786, 0.796, 0.832, and 0.815, respectively (Figures 6B–D). Since TNM staging is conventionally used to assess prognosis and guide treatment strategies for ICC patients, the predictive accuracy of the developed nomograms was compared to TNM staging using time-dependent ROC (td-ROC) analysis. The results demonstrated that the nomograms significantly outperformed TNM staging in prognostic prediction for both OS and RFS (Figures 5E, 6E). Collectively, the constructed nomograms, incorporating GNRI and body composition metrics, demonstrated superior prognostic efficiency compared to the TNM staging system, offering a more accurate tool for risk stratification in elderly ICC patients.

**Table 4:** Accuracy of nomogram and TNM stage in predicting survival for ICC patients.

	Models	C-index	AUC of 1 year	AUC of 3 years	AUC of 5 years	Integrate AUC
OS	Nomogram	0.734	0.805	0.786	0.843	0.809
	8th TNM stage	0.542	0.548	0.578	0.614	0.581
RFS	Nomogram	0.704	0.786	0.796	0.832	0.815
	8th TNM stage	0.535	0.591	0.568	0.615	0.581

ICC, intrahepatic cholangiocarcinoma; TNM, tumor-node-metastasis; OS, overall survival; RFS, recurrence-free survival; AUC, area under the curve.

## Discussion

Over the past two decades, the proportion of individuals aged over 65 has increased rapidly, accounting for approximately 10% of the global population. Aging is associated with a progressive decline in muscle mass, function, and quality, particularly among those aged 65 and older. In this study, we conducted a comprehensive evaluation of body composition and its impact on long-term survival in a large-scale retrospective cohort of 157 elderly ICC patients. Our findings demonstrated that low GNRI, sarcopenia, visceral adiposity, and IMF deposition serve as complementary risk factors for poor prognosis, with patients exhibiting these indicators experiencing significantly worse OS and RFS. Furthermore, we developed a prognostic nomogram based on independent risk factors and validated its strong predictive performance. Notably, our nomogram outperformed the conventional TNM staging system in prognostic assessment, providing a more accurate tool for risk stratification in elderly ICC patients.

Clinical estimation of body composition using conventional parameters, such as body weight, waist circumference, BMI, physical activity, and handgrip strength, remains challenging and is often compromised by a patient’s physical condition (5). In this study, we comprehensively analyzed the impact of GNRI combined with body composition on survival in a cohort of elderly ICC patients and identified skeletal muscle index (SMI), muscle attenuation (MA), and visceral-to-subcutaneous adipose tissue area ratio (VSR) as independent risk factors for poor prognosis. In addition, we examined the relationships among these body composition parameters and found that elderly ICC patients with a greater number of adverse body composition components had significantly worse survival outcomes.

A recent global cancer trend report highlighted that population aging contributed to the largest increase in cancer incidence, accounting for 56.5% (4.7 million) of cases and 63.3% (3.6 million) of deaths between 1990 and 2019 (15). According to our previous study, 26.1% of ICC patients who underwent curative resection were elderly (11). Older patients generally have a worse prognosis, particularly in cancer, due to their heightened vulnerability to malnutrition and immune dysfunction (16). Given these factors, GNRI and body composition parameters may serve as powerful prognostic indicators for ICC in elderly patients.

Malnutrition is prevalent in elderly patients, particularly those with malignancies (17). GNRI is an objective screening tool that predicts nutrition-related morbidity and mortality risks without the influence of underlying inflammatory processes (18). Since its introduction by Bouillanne in 2005, GNRI has been widely used to assess postoperative complications and overall survival in oncologic patients (19). A recent study by Riveros et al. confirmed that GNRI is a standardized nutritional screening tool capable of preoperatively assessing the risk of complications in elderly patients undergoing radical cystectomy (20). Consistent with our findings, low GNRI scores were significantly associated with worse survival outcomes in elderly ICC patients who underwent surgery.

Sarcopenia, a syndrome characterized by progressive declines in skeletal muscle mass (low SMI) and quality (low MA), can be classified as either primary or secondary (21). Primary sarcopenia results from age-related muscle loss, whereas secondary sarcopenia is linked to chronic diseases such as cancer and infections (10). Interestingly, Fujiwara et al. suggested that functional age, rather than chronological age, is more critical in determining prognosis in HCC patients, indicating that sarcopenia is not solely age-dependent but also influenced by metabolic status (5). Low muscle mass is associated with insulin resistance, systemic inflammation, and immune senescence, all of which contribute to tumor progression (22). In addition, muscle attenuation (myosteatosis) is characterized by inter- and intra-myocellular fat accumulation, which creates a pro-inflammatory microenvironment that weakens immune function via cytokines (23, 24). Myosteatosis has been linked to oxidative stress, reduced muscle function, and poor perioperative outcomes (25). Several studies have demonstrated that low muscle quality correlates with reduced muscle strength and poor survival outcomes in patients undergoing surgery for colorectal or head and neck cancer (26, 27). Similarly, we previously reported that low SMI is significantly associated with poor survival in ICC patients (11). Our current analysis further confirms that in elderly ICC patients, both low SMI and low MA negatively impact survival outcomes.

With aging, adipose tissue shifts from subcutaneous to visceral depots and ectopic sites, leading to altered metabolic and inflammatory profiles. Compared to subcutaneous adipose tissue (SAT), visceral adipose tissue (VAT) is more vascular, innervated, and metabolically active, with a higher number of inflammatory and immune cells. VAT is also more insulin-resistant and secretes adipokines that trigger hepatic immune activation, leading to inflammation. There are more glucocorticoid and androgen receptors in VAT than in SAT. VAT adipocytes are more metabolically active, more sensitive to lipolysis, and more insulin-resistant than SAT adipocytes (28). In addition, VAT venous blood is drained directly to the liver through the portal vein. This contrasts with SAT where venous drainage is through systemic veins. The portal drainage of visceral fat provides direct hepatic access to free fat acids and adipokines secreted by visceral adipocytes. Adipokines activate hepatic immune mechanisms with the production of inflammatory mediators such as C-reactive protein (28). These differences may explain how body fat distribution influences immune function, inflammation, and endocrine homeostasis, ultimately affecting disease prognosis. Numerous studies have reported that higher VAT levels are associated with impaired surgical outcomes and poor long-term survival (5). In this study, we identified a high visceral-to-subcutaneous adipose tissue area ratio (VSR) as a significant predictor of worse survival outcomes in elderly ICC patients.

Aging inevitably leads to declining muscle mass, impaired muscle function, and fat redistribution. Interestingly, myosteatosis can develop independently of muscle mass but may have a synergistic effect, exacerbating functional impairment. Recent evidence suggests that fat accumulation within muscle can negatively affect physiological responses, even in individuals with normal muscle mass (29). Despite these complexities, our study found a statistically significant relationship between muscle loss, fat redistribution, and poor prognosis in elderly ICC patients. At the molecular level, aging disrupts the balance between protein synthesis and proteolysis, as well as fat metabolism, further exacerbating malnutrition (30). These alterations create a vicious cycle, impairing muscle regeneration and accelerating muscle degradation (31). Accordingly, our findings confirm that elderly ICC patients with multiple adverse body composition parameters tend to have significantly worse survival outcomes. Our prognostic nomogram can assist in preoperative risk stratification, guiding patient selection for surgical resection or alternative treatments. High-risk patients, particularly those with low GNRI or sarcopenia, may benefit from prehabilitation strategies, such as nutritional interventions and resistance training, to improve surgical outcomes. In addition, these patients may require closer postoperative surveillance with more frequent imaging and tumor marker monitoring to detect early recurrence.

Despite its strengths, this study has several limitations. First, we focused only on elderly patients (aged 65 and older) with a relatively small sample size. While sarcopenia and visceral adiposity are more pronounced in elderly individuals due to age-related muscle loss and fat redistribution, metabolic and inflammatory changes related to poor body composition may also affect ICC prognosis in younger patients (15). Future studies should explore age-specific cutoffs for body composition parameters. In addition, incorporating machine learning and deep learning approaches could improve predictive accuracy in larger datasets. Second, as a retrospective study, some clinical data were incomplete, and patients without imaging data were excluded, potentially leading to selection bias. Moreover, while our study is based on multi-institutional data, variations in surgical techniques and postoperative management across centers may affect the generalizability of our findings. External validation in independent cohorts is essential to confirm these results.

## Conclusion

The present study suggested that GNRI, sarcopenia, IMF deposition, and visceral adiposity independently predicted mortality and tumor recurrence in elderly ICC patients following curative liver resection. Body composition was a major determinant of prognosis in patients with ICC. Furthermore, we established a prognostic nomogram based on GNRI and body composition revealed superior prognostic efficacy over conventional TNM stages. In the future, interventions targeting body composition, such as exercise therapy, nutritional support, and even the use of drugs to prevent muscle depletion, may be effective in improving the long-term survival of ICC patients, especially for the elderly.

## Data Availability

The original contributions presented in the study are included in the article/Supplementary material, further inquiries can be directed to the corresponding authors.

## Footnotes

^1^ http://www.R-project.org

## Supplementary material

SUPPLEMENTARY FIGURE S1 Flow diagram showing study design. The multi-institutional dataset was went through to include patients. A total of 157 elderly patients passed the exclusion criteria were identified.

SUPPLEMENTARY FIGURE S2 Kaplan-Meier curves showed overall survival **(A)** and recurrence-free survival **(B)** according to BMI. BMI was classified as less than was classified as underweight (<20kg/m_2_), normal weight, and overweight (> 24kg/m_2_). BMI, body mass index. P value was determined by log-rank analysis.

SUPPLEMENTARY FIGURE S3 The correlations between BMI and GNRI **(A)**, SMI **(B)**, VSR **(C)**, MA **(D)**. BMI was weakly correlated to GNRI, although statistically significant. BMI, body mass index; GNRI, geriatric nutritional risk index; SMI, skeletal muscle index; VSR, visceral to subcutaneous adipose tissue area ratio; MA, muscle attenuation.

SUPPLEMENTARY FIGURE S4 The correlations between GNRI and SMI **(A)**, VSR **(B)**, MA **(C)** were statistically significant. GNRI, geriatric nutritional risk index; SMI, skeletal muscle index; VSR, visceral to subcutaneous adipose tissue area ratio; MA, muscle attenuation.

SUPPLEMENTARY FIGURE S5 Definitions and classifications of the GNRI and prognostic body composition components. The stratification was based on gender. GNRI, geriatric nutritional risk index; SMI, skeletal muscle index; VSR, visceral to subcutaneous adipose tissue area ratio; MA, muscle attenuation.
