# Supplementary figures and images for: Geriatric nutritional risk index and body composition dictate the prognosis of elderly patients with intrahepatic cholangiocarcinoma
Source: Front Nutr. 2025 Mar 7;12:1565317. doi: 10.3389/fnut.2025.1565317 (PMC11925769; doi:10.3389/fnut.2025.1565317)

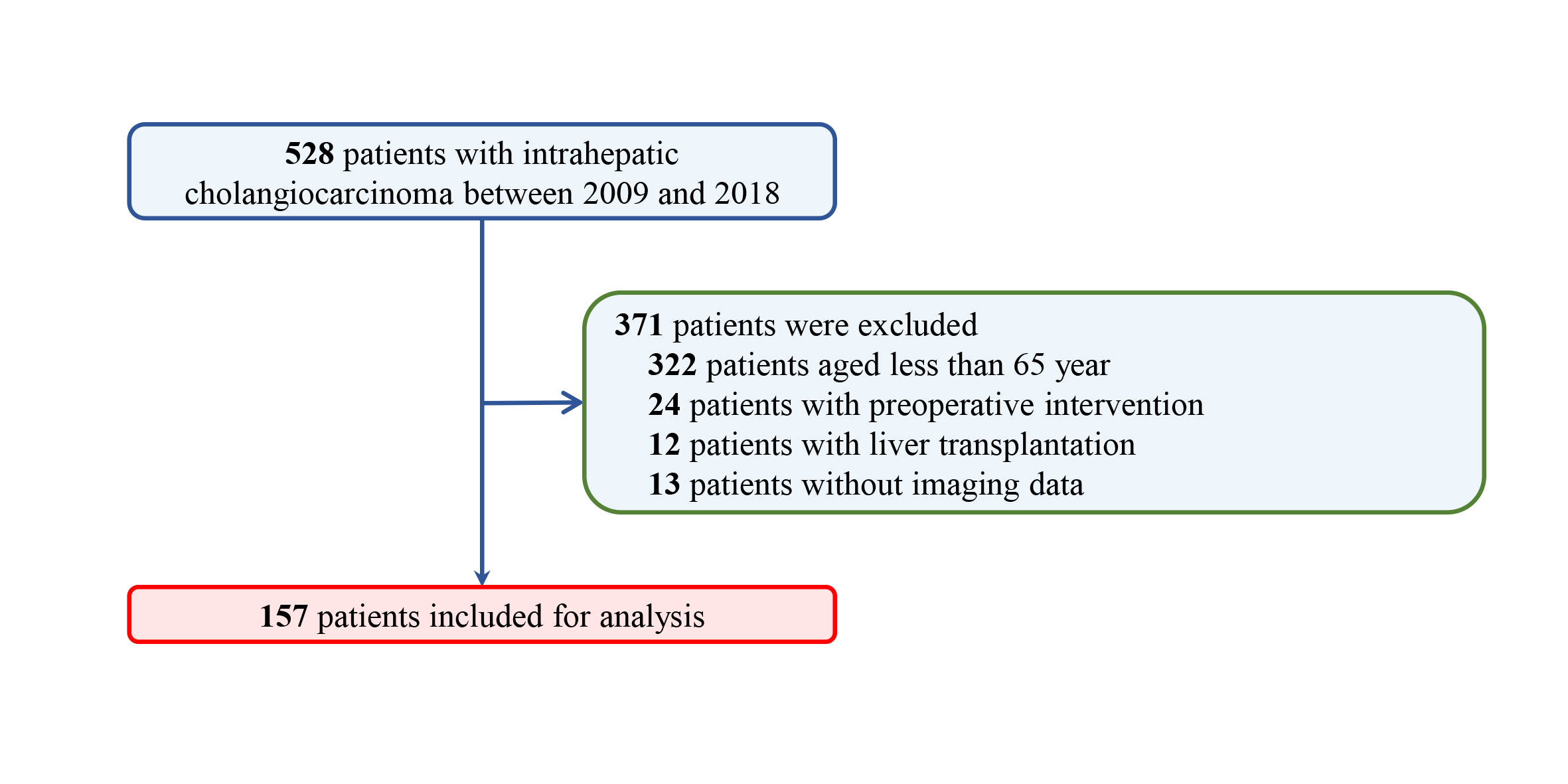

Supplement: SUPPLEMENTARY FIGURE S1 — Flow diagram showing study design. The multi-institutional dataset was went through to include patients. A total of 157 elderly patients passed the exclusion criteria were identified. [file Image_1.jpeg]

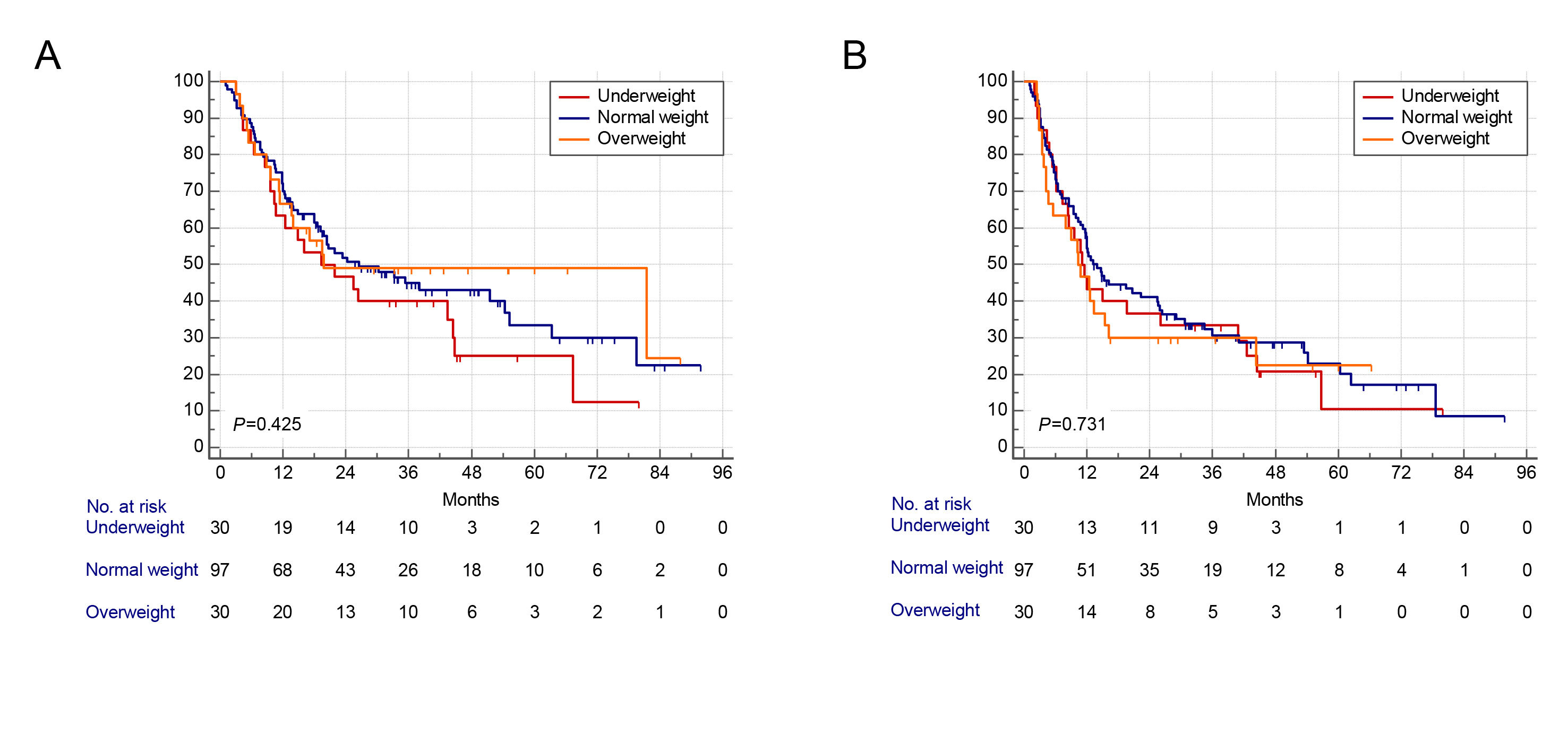

Supplement: SUPPLEMENTARY FIGURE S2 — Kaplan-Meier curves showed overall survival (A) and recurrence-free survival (B) according to BMI. BMI was classified as less than was classified as underweight (<20kg/m2), normal weight, and overweight (> 24kg/m2). BMI, body mass index. P value was determined by log-rank analysis. [file Image_2.jpeg]

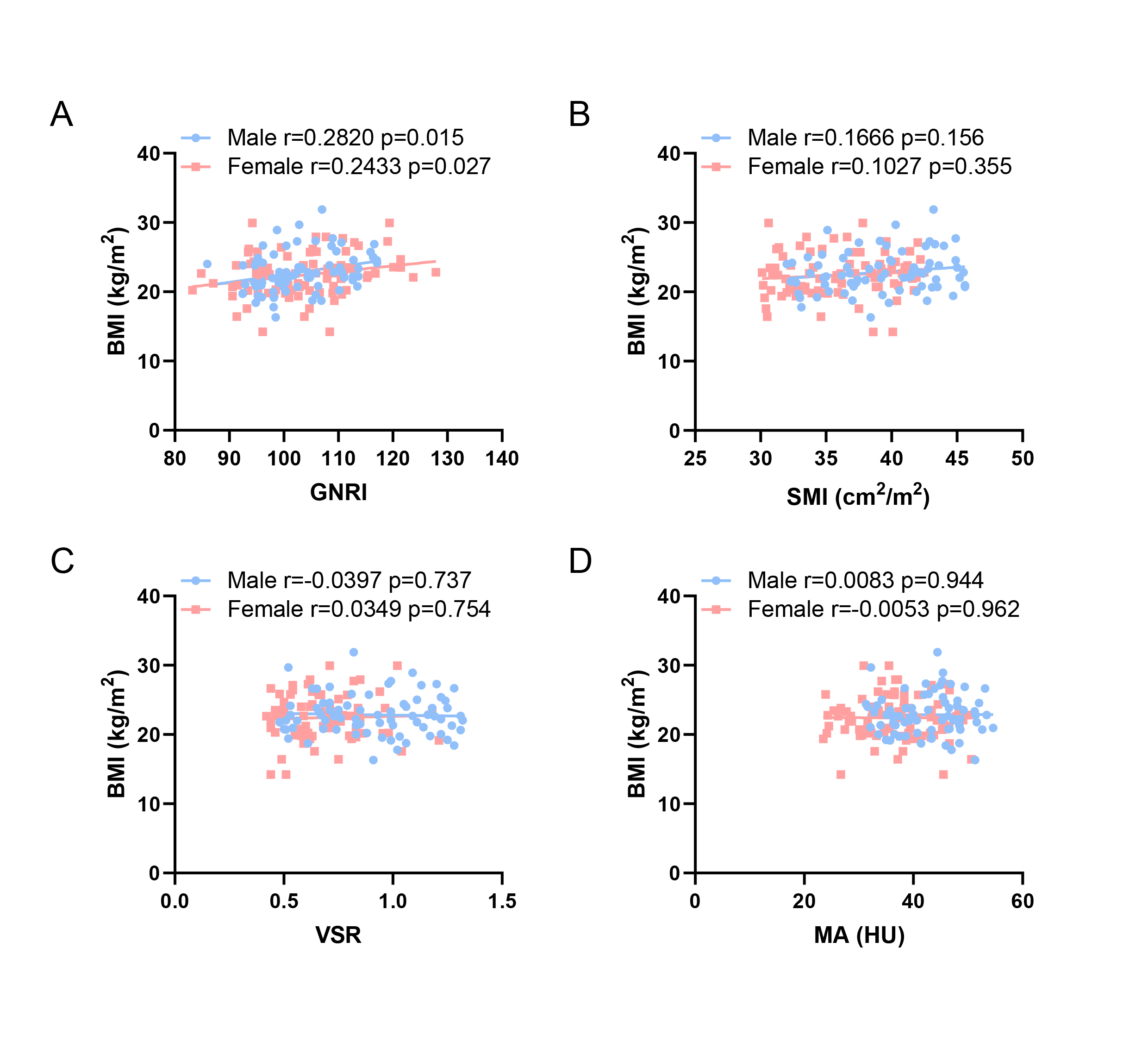

Supplement: SUPPLEMENTARY FIGURE S3 — The correlations between BMI and GNRI (A), SMI (B), VSR (C), MA (D). BMI was weakly correlated to GNRI, although statistically significant. BMI, body mass index; GNRI, geriatric nutritional risk index; SMI, skeletal muscle index; VSR, visceral to subcutaneous adipose tissue area ratio; MA, muscle attenuation. [file Image_3.jpeg]

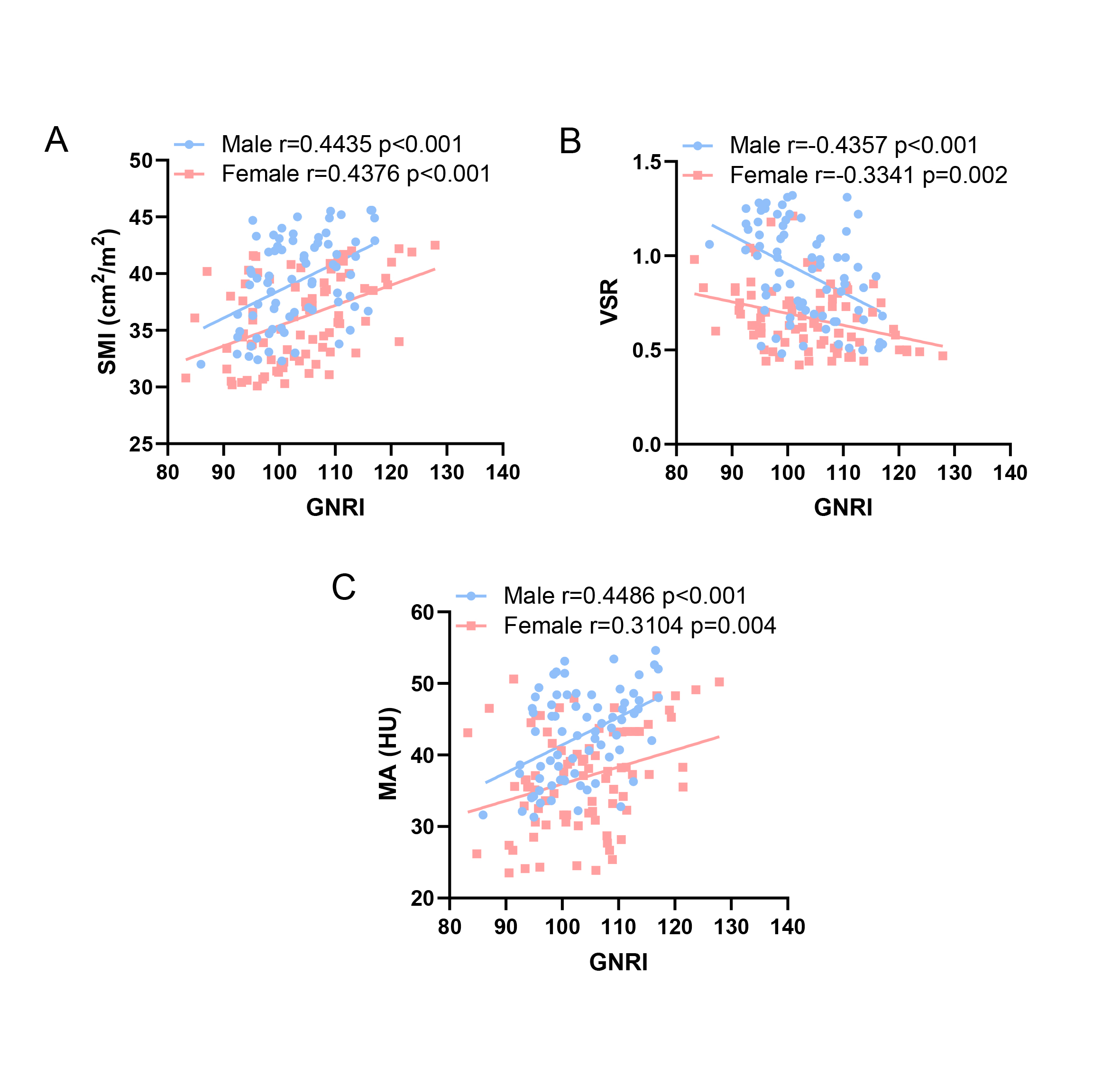

Supplement: SUPPLEMENTARY FIGURE S4 — The correlations between GNRI and SMI (A), VSR (B), MA (C) were statistically significant. GNRI, geriatric nutritional risk index; SMI, skeletal muscle index; VSR, visceral to subcutaneous adipose tissue area ratio; MA, muscle attenuation. [file Image_4.jpeg]

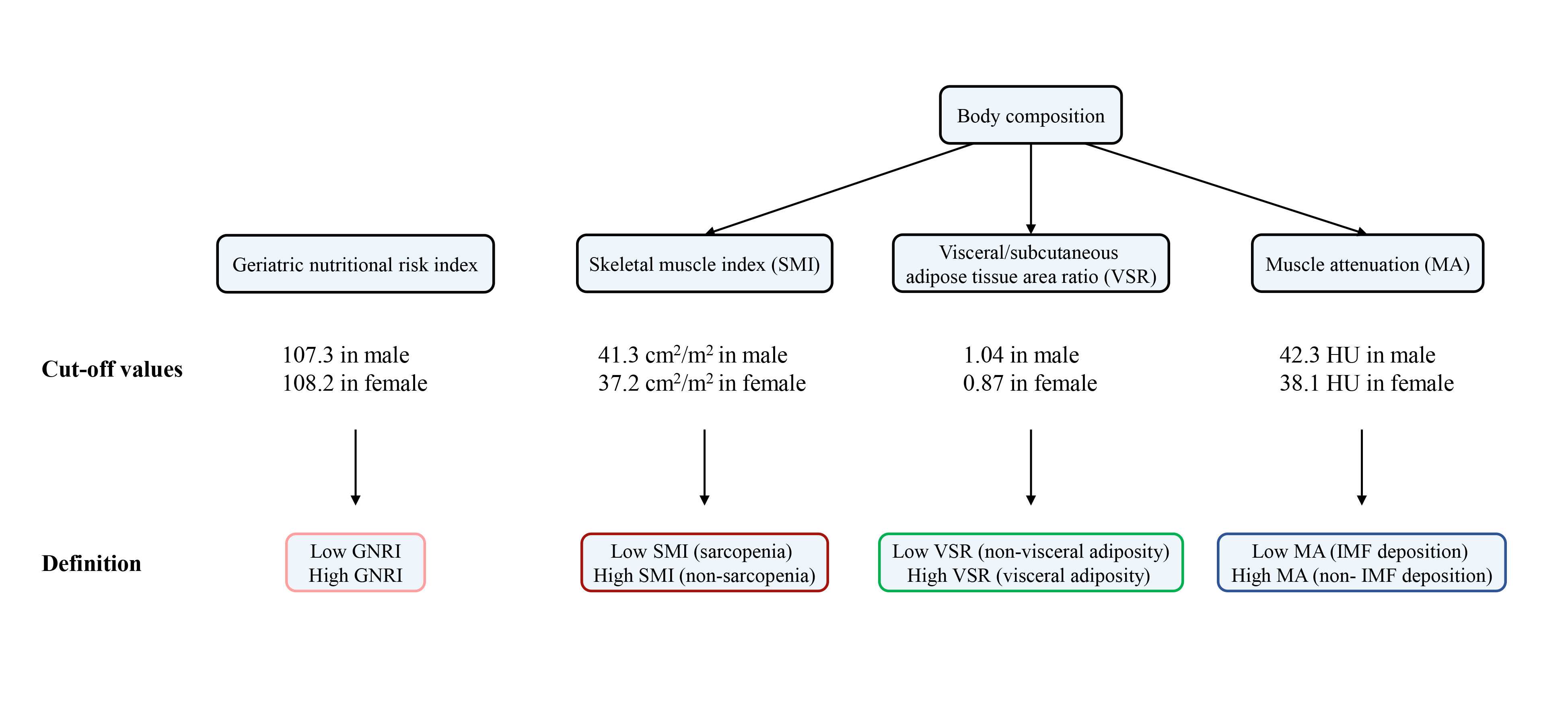

Supplement: SUPPLEMENTARY FIGURE S5 — Definitions and classifications of the GNRI and prognostic body composition components. The stratification was based on gender. GNRI, geriatric nutritional risk index; SMI, skeletal muscle index; VSR, visceral to subcutaneous adipose tissue area ratio; MA, muscle attenuation. [file Image_5.jpeg]
